# Supplementary material for: ChatGPT as a Tool for Biostatisticians: A Tutorial on Applications, Opportunities, and Limitations
Source: Stat Med. 2025 Oct 23;44(23-24):e70263. doi: 10.1002/sim.70263 (PMC12548020; doi:10.1002/sim.70263)
Supplement: Supplementary file 1 — Data S1. Supporting Information. [file SIM-44-0-s001.zip › Supplementary Materials Additional.zip!/3.1_Systematic_Reviews_and_Meta_aspects/3.1_Data Extraction - o4-mini - Chat 1.pdf]

Use Case Meta-Analysis - Data Extraction - o4-mini - Chat 1

Vidali et al. - 2012 - Conservative treatment of breast ductal carcinoma in situ results of an Italian multi-institutional.pdf
PDF

Rudloff et al. - 2009 - Concurrent lobular neoplasia increases the risk of ipsilateral breast cancer recurrence in situ treated with breast-conserving therapy.pdf
PDF

The aim is to perform a meta-analysis on total ipsilateral local recurrence rates at 10 years in cases of ductal carcinoma in situ.

Data inclusion rules are: To ensure the validity of the follow-up data, the results should be as close to 10 years follow-up (minimum) as the information in each study allows. If there is more than one publication from an institution or group, the most recent study with appropriate follow-up will be used to extract 10-year data.

Please provide from each of the two attached studies:

- the rate of ipsilateral local recurrence for conservative surgery with additional radiotherapy (no other interventions or drugs allowed) after 10 years

- the number of eligible patients

- period of data collection

- duration of follow-up

- type of study

- age of patients (mean or median, maximum and minimum)

- treatment modalities

For this task you have access to up-to-the-minute web retrieval to find adequate approaches for data extraction. When answering the task above please proceed as follows:

1. Fetch the most relevant sources.

2. Show your step-by-step reasoning.

3. At the end, give me a concise answer citing the sources you used.

4. Please provide a dataframe with one entry for each study, and column names "study", "rate", "n_patients", "period", "follow_up", "study_type", "age", and "treatment".

Step-by-step reasoning:

1. **Identify relevant studies**
    - Vidali et al. (2012) – a multi-institutional retrospective Italian study of DCIS treated with breast-conserving surgery (BCS) + radiotherapy (RT) from February 1985 to March 2000 .
    - Rudloff et al. (2009) – a single-institution retrospective cohort of DCIS treated with breast-conserving therapy (surgery + RT) between 1991 and 1995 .
2. **Extract key data for the meta-analysis inclusion criteria**
    - **Eligibility** (number of patients and data collection period)
    - **Follow-up** (duration and whether ≥10 years)
    - **Local recurrence rate at 10 years** (ipsilateral)
    - **Study type** (retrospective vs. prospective; single vs. multi-institutional)
    - **Patient age** (median and range)
    - **Treatment modalities** (surgery + RT specifics; any additional drugs)
3. **Confirm each datum in the papers**
    - **Vidali et al.**
        - 586 patients treated Feb 1985–Mar 2000; median follow-up 136 months (11.3 years; range 16–292) .
        - 10-year actuarial local recurrence rate: 9.6% .
        - Median age 55 years (range 29–84).
        - Treatment: BCS (quadrantectomy/tumorectomy/wide excision) + whole-breast RT (median 50 Gy), with boost in 50% (median 10 Gy).
    - **Rudloff et al.**
        - 294 patients treated 1991–1995; median follow-up 11 years (range 0–16) .
        - 10-year cumulative IBTR rate: 22% .
        - Median age 55 years (range 26–89).
        - Treatment: BCS + external-beam RT; 6.1% received tamoxifen post-DCIS diagnosis .
4. **Compile a concise summary and present as a dataframe**

Concise answer:

study	rate (10 yr ipsi. recurrence)	n_patients	period	follow_up	study_type	age	tr
Vidali et al. (2012)	9.6%	586	Feb 1985–Mar 2000	median 136 mo (16–292)	retrospective, multi-institutional (10 centers)	median 55 y (range 29–84 y)	BCS + w RT (me

study	rate (10 yr ipsi. recurrence)	n_patients	period	follow_up	study_type	age	tr
							boost i 10 Gy)
Rudloff et al. (2009)	22%	294	1991–1995	median 11 y (0–16)	retrospective, single-institution cohort	median 55 y (range 26–89 y)	BCS + e RT; tan

Quellen
